# Involvement of CD147 in overexpression of MMP-2 and MMP-9 and enhancement of invasive potential of PMA-differentiated THP-1

**DOI:** 10.1186/1471-2121-6-25

**Published:** 2005-05-17

**Authors:** Jun Zhou, Ping Zhu, Jian Li Jiang, Qing Zhang, Zhen Biao Wu, Xi Ying Yao, Hao Tang, Ning Lu, Yong Yang, Zhi Nan Chen

**Affiliations:** 1 Department of Clinical Immunology, First Affiliated Hospital, Fourth Military Medical University, 17 Changlexilu, Xi'an 710032, Shaanxi, P.R. of China; 2 Department of Cell Biology / Cell Engineering Research Center, Fourth Military Medical University, 17 Changlexilu, Xi'an 710032, Shaanxi, P.R. of China; 3 Department of pharmacology, School of Medicine of Xi'an Jiaotong University, Xi'an 710061, Shaanxi, P.R. of China

## Abstract

**Background:**

During infection and inflammation, circulating blood monocytes migrate from the intravascular compartments to the extravascular compartments, where they mature into tissue macrophages. The maturation process prepares the cells to actively participate in the inflammatory and immune responses, and many factors have been reported to be involved in the process. We found in our study that CD147 played a very important role in this process.

**Results:**

By using PMA-differentiated human monocyte cells line THP-1, we found that CD147 mediated matrix metalloproteinases (MMPs) expression of the leukemic THP-1 cells and thus enhanced the invasiveness of THP-1 cells. After 24 hours of PMA-induced monocyte differentiation, the mean fluorescence intensity of CD147 in differentiated THP-1 cells (289.61 ± 31.63) was higher than that of the undifferentiated THP-1 cells (205.1 ± 19.25). There was a significant increase of the levels of proMMP-2, proMMP-9 and their activated forms in the differentiated THP-1 cells. Invasion assays using reconstituted basement membrane showed a good correlation between the invasiveness of THP-1 cells and the production of MMP-2 and MMP-9. The difference in the MMPs expression and the invasive ability was significantly blocked by HAb18G/CD147 antagonistic peptide AP-9. The inhibitory rate of the secretion of proMMP-9 in the undifferentiated THP-1 cells was 45.07%. The inhibitory rate of the secretion of proMMP-9, the activated MMP-9 and proMMP-2 in the differentiated THP-1 cells was 52.90%, 53.79% and 47.80%, respectively. The inhibitory rate of invasive potential in the undifferentiated cells and the differentiated THP-1 cells was 41.82 % and 25.15%, respectively.

**Conclusion:**

The results suggest that the expression of CD147 is upregulated during the differentiation of monocyte THP-1 cells to macrophage cells, and CD147 induces the secretion and activation of MMP-2 and MMP-9 and enhances the invasive ability of THP-1 cells. The matured monocytes / macrophages, via their high expression of CD147, may play an important role in promoting the tissue repair or tissue damage during their inflammatory response.

## Background

Activated macrophages are known to play an important role in the degradation process of normal and abnormal matrix. Upon differentiation, the response of macrophages to pathogens is markedly enhanced, allowing them to participate in the inflammatory and immune responses [1]. The differentiation process is a complex one and is controlled by the expression or the activation of several factors [2]. The activated inflammatory macrophage plays a crucial role in matrix destruction by producing matrix metalloproteinases (MMPs) both directly and indirectly [3-5]. Activated macrophages up-regulate the activity of MMPs, particularly that of MMP-9, MMP-2 (gelatinases), MMP-12 (metalloelastase) and MMP-7 (matrilysin) [6,7]. Activated macrophages are also known to release collagenases (MMP-1 and MMP-13) under certain circumstances [8]. In co-culture assays, macrophages can stimulate myofibroblasts to release collagenases. In the studies of kidney disease, inflammatory macrophages of the glomeruli are found to have induced myofibroblast mesangial cells to produce stromelysin (MMP-3) [9].

MMPs are a family of structurally related endopeptidases that resorb macromolecules of the extracellular matrix. They participate both in physiologic connective tissue remodeling and in pathologic tissue destruction. However, the exact mechanism underlying the up-regulated expression of MMPs in macrophages is not yet clear.

CD147, a 57-KD transmembrane glycoprotein, (also called extracellular matrix metalloproteinase inducer (EMMPRIN) and leukocyte activation-associated M6 antigen) is located on the surface of human tumor cells and normal keratinocytes [10,11]. CD147, rich on the surface of most tumor cells, has been found to stimulate tumor cells and stromal cells to produce elevated levels of MMPs [12-16]. However, the involvement of CD147 in stimulating MMP release and activation during cellular maturation process remains unknown. This paper reports our study, using a phorbol myristate acetate (PMA)-induced cell differentiation model of the human monocytic cell line THP-1 [17], on the involvement of CD147 expression in the differentiation process of monocytes into macrophages, and on the correlation between CD147 expression and the secretion and activation of MMP-2 and MMP-9, and the correlation between CD147 expression and the invasive ability of mono-macrophage cells.

## Results

### Alteration of morphology and phenotype on PMA-treatmented THP-1 cells

**Figure 1:**
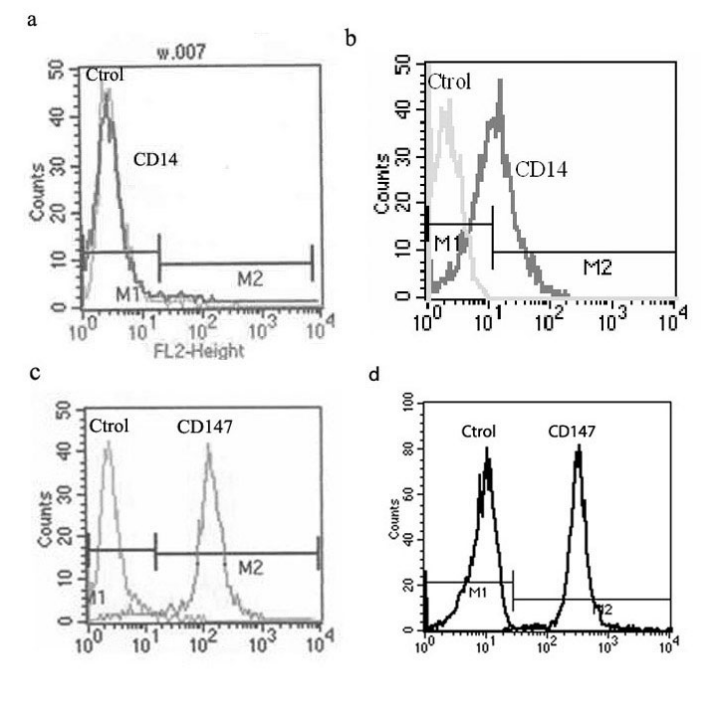
Expression of CD14 and CD147 on the surface of differentiated and undifferentiated THP-1 cells. THP-1 cells were incubated for 24 h in the presence (b and d) or absence (a and c) of PMA (200 uM). Expression of CD14 and CD147 were measured by flow cytometry using FITC-labeled anti-CD14 mAb and anti-CD147 mAb. Analysis was conducted on a FACS™ brand flow cytometer.

Untreated THP-1 cells are round in shape and do not adhere to the plastic surfaces of the culture plates. In the presence of 200 nM PMA for 24 h, the cells became flat and amoeboid in shape, and adhered to the dish bottom. Flow cytometry analysis revealed that these cells expressed higher levels of CD14 (51.19%), a macrophage-specific differentiation antigen, than those (2.55%) of the untreated THP-1 cells (p < 0.05) (Figure 1a, 1b). The mean fluorescence intensity (MFI) of CD147 in the differentiated THP-1 cells (289.61 ± 31.63) was higher than that of the undifferentiated THP-1 cells (205.1 ± 19.25) (p < 0.05) (Figure 1c, 1d).

### MMPs release, activation and invasive ability of differentiated THP-1 cells

**Figure 2:**
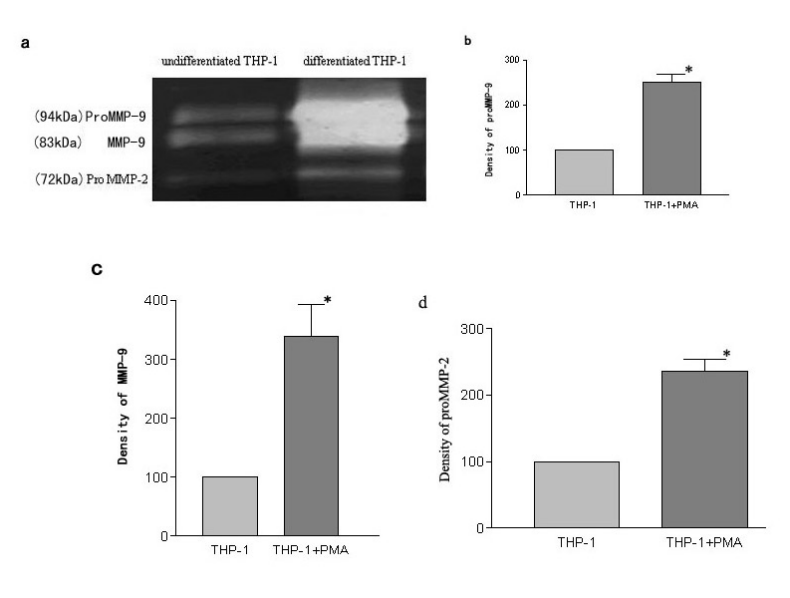
Gelatin zymography of culture medium conditioned by undifferentiated THP-1 cells and differentiated THP-1 cells. Cells were cultured in serum-free medium for 5–20 h, and the conditioned media were collected and analyzed for MMP activity by gelatin zymography. (a) image of gelatin zymography. Top two bands correspond to MMP-9 gelatinase, lower two bands to MMP-2 gelatinase. (b), (c), (d) Densitometry analysis of MMP activity. The value of MMP activity of undifferentiated THP-1 cells is shown as control (100%). Mean ± SE of at least 6 independent samples per group. p < 0.05, Student's t test.

**Figure 3:**
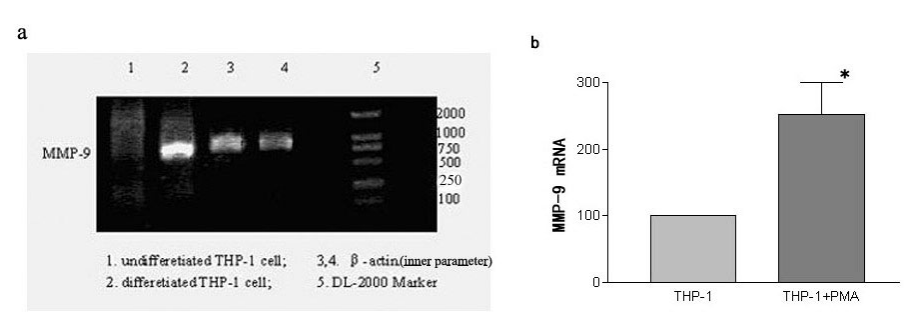
RT-PCR analysis of MMP-9 mRNA expression in human macrophages after 24-hour PMA treatment. THP-1 cells were treated with 100 μg/ml PMA for 24 h, and total RNA was isolated by electrophoresis and transferred to a nylon membrane. The membrane was hybridized with MMP-9 specific cDNAs, and visualized by autoradiography. (a) nuclear run-on assay for newly transcribed undifferentiated and differentiated THP-1 cell mRNA. β-actin was used as the inner parameter; (b) Densitometric analysis of the bands in panel A. MMP-9 mRNA of undifferentiated THP-1 cells is shown as control (100%). Mean ± SE of at least 6 independent samples per group. p < 0.05, Student's t test.

**Figure 4:**
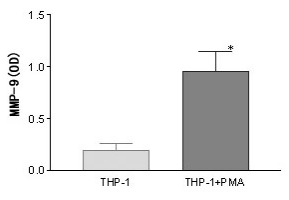
ELISA assay MMP-9 secretion in undifferentiated and differentiated THP-1 cells.

**Figure 5:**
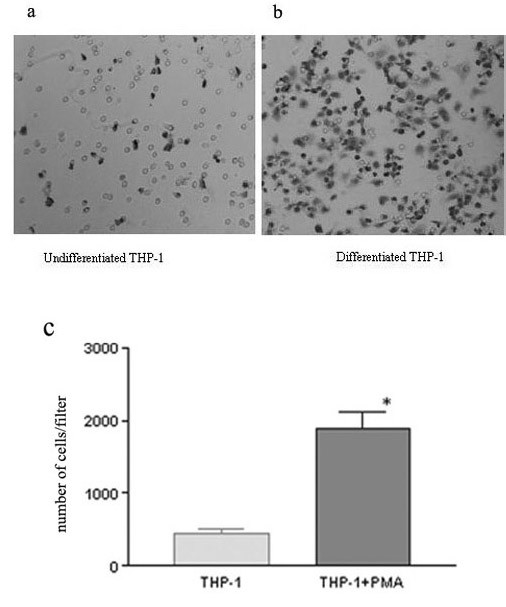
The invasive potential of undifferentiated and differentiated THP-1 cells. The invasive potential was evaluated in Transwell chambers as described in Materials and Methods. Briefly, cells were suspended in serum-free medium supplemented with or without PMA (100 uM) and seeded into the upper side of the Matrigel (5 μg/ml)-coated chambers. After incubation for 24 h at 37°C, the number of invaded cells was determined using a colorimetric crystal violet assay. Values are the means ± SE (n = 3~4).

As shown in Figure 2, SDS-polyacrylamide gelatin electrophoresis zymography showed that the secretion and activation of MMP-2 and MMP-9 were significantly enhanced in differentiated THP-1 cells compared with those observed in the undifferentiated THP-1 cells (p < 0.05). RT-PCR showed that MMP-9 mRNA significantly increased after a 24-hour PMA treatment (Figure 3). As shown in Figure 4, a significantly increase was observed by ELISA in the release of MMP-9 in the differentiated THP-1 cells compared with that in the undifferentiated THP-1 cells. The differentiated THP-1 cells, after a 24-hour PMA treatment, were found to have significantly higher number of cells/filter to invade through transwell chambers (1626 ± 476.8) than that (282.3 ± 74.87) of undifferentiated THP-1 cells (p < 0.05) (Figure 5).

### Effect of HAb18G/CD147 antagonistic peptide AP-9 on MMPs release and activation and Invasion processes

**Figure 6:**
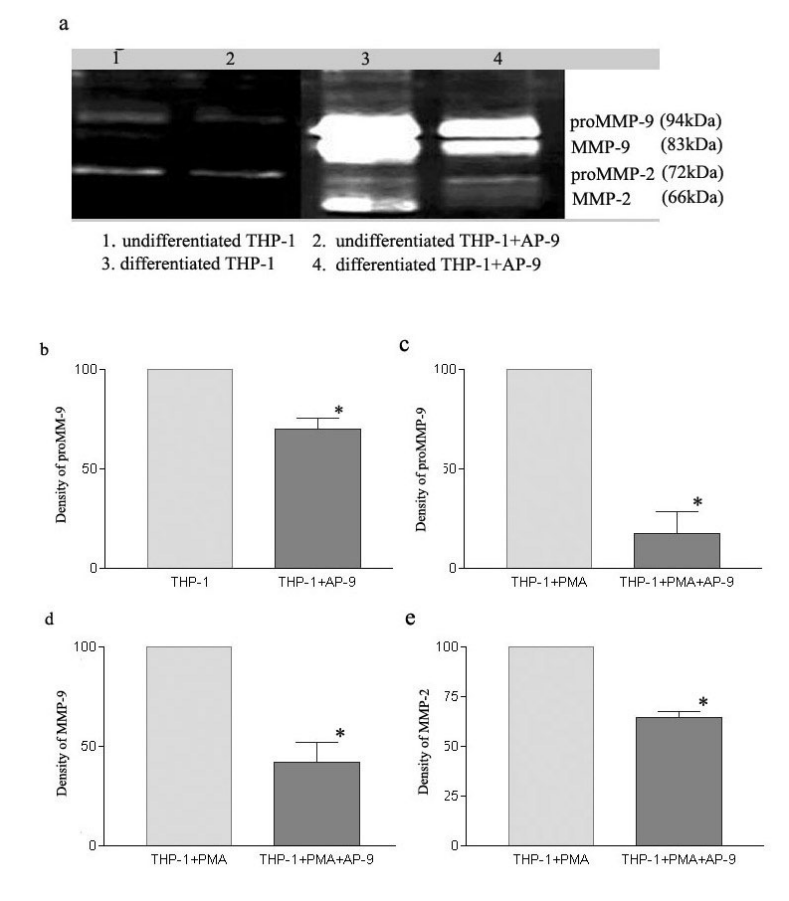
Effects of AP-9 on MMPs release and activation in undifferentiated and differentiated THP-1 cells. (a) Image of gelatin zymography. Top two bands correspond to MMP-9 gelatinase, lower two bands to MMP-2 gelatinase. (b), (c), (d), (e) Densitometry analysis of MMP activity. The value of MMP activity of THP-1 cells without AP-9 is shown as control (100%). Mean ± SE of at least 6 independent samples per group. p < 0.05, Student's t test. (b) Effects of AP-9 on pro-MMP-9 activity of undifferentiated THP-1 cells. (c) Effects of AP-9 on pro-MMP-9 activity of differentiated THP-1 cells. (d) Effects of AP-9 on MMP-9 activity of differentiated THP-1 cells. (e) Effects of AP-9 on MMP-2 activity of differentiated THP-1 cells.

A significantly reduced release and activation of MMP-9 and MMP-2 from cells pre-incubated with HAb18G/CD147 antigonistic peptide AP-9 were found by gelatin zymography. The inhibitory rate of the secretion of proMMP-9 in the undifferentiated THP-1 cells was 45.07%. The inhibitory rate of the secretion of proMMP-9, the activated MMP-9 and proMMP-2 in differentiated THP-1 cells was 52.90%, 53.79% and 47.80%, respectively (p < 0.05) (Figure 6a, 6b, 6c, 6d, 6e).

**Figure 7:**
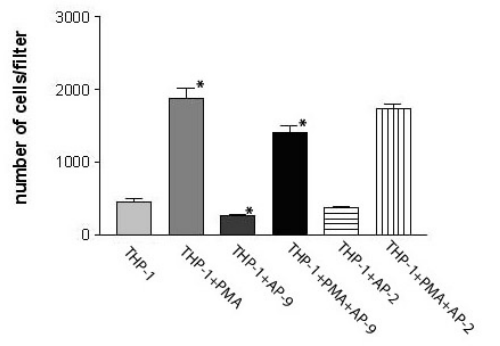
Inhibitory effects of AP-9 on invasive potential of undifferentiated and differentiated THP-1 cells to Matrigel. Cells were suspended in serum-free medium and seeded into the matrigel (5 ug/ml)-coated wells. After the incubation for 24 h at 37°C, the number of invaded cells was determined using a colorimetric crystal violet assay. Values are the means ± SE (n = 3~4).

Invasion assay showed that the amounts of cells invaded through Matrigel coated filter decreased after the treatment with AP-9 (200 ug/ml) for 24 h in both undifferentiated and differentiated THP-1 cells. The inhibitory rate of invasive potential in the undifferentiated cells and the differentiated THP-1 cells was 41.82 % and 25.15%, respectively (p < 0.05) (Figure 7), control peptide SSP was not inhibitory.

## Discussion

In this study we demonstrate that the overexpression of CD147 enhances the release and the activation of MMPs (MMP-2 and MMP-9) and the invasive potential during the differentiation of monocyte THP-1 cells to macrophage cells. This is in accordance with our previous study in human hepatoma cells [18].

MMPs are a family of Zn^2+^-containing enzymes that cleave most of the components of extracellular matrix (ECM) and are involved in physiologic connective tissue remodeling and in pathologic tissue destruction. MMPs can be regulated by different factors. Popp et al reported that calpain/calpastatin system mediated MMP-mRNA expression of the leukemic THP-1 cells and, as a result, their invasiveness [19]. Worley et al identified that PPARγ and RXR agonists had complex effects on monocyte MMPs expression [20]. In our present study, we employed CD147 to promote MMPs.

CD147 is a highly glycosylated transmembrane protein belonging to the immunoglobulin superfamily with two Ig domains. Previous studies have clearly demonstrated that CD147 is highly expressed on some tumor cells and is responsible for stimulating MMP production by stromal cells and/or other tumor cells, thereby leading to extracellular matrix degradation and elevated tumor growth and metastasis [21,22]. CD147 has also been found to stimulate the secretion and the activation of MMPs, which are associated with tissue degradation and remodeling during inflammatory damage and wound healing. The up-regulation of the expression of CD147 in the synovial membrane of rheumatoid arthritis (RA) patients has been reported [23,24]. Our recent studies have demonstrated that CD147 is highly expressed on monocytes in circulating blood and synovial fluid, and on macrophages/macrophages-like synovial cells in synovium from RA patients [25]. Similarly, the up-regulation of the expression of CD147 on monocytes/macrophages may induce MMPs produced by fibroblasts and other monocytes/macrophages, and this process may play an essential role in articular cartilage lesion development in RA.

To explore the association of CD147 with the secretion and activation of MMPs, especially those in the cellular maturation, in our present study, human THP-1 monocytic cells were further stimulated to differentiate into a macrophage-like stage and some specific morphological and phenotypic alterations of PMA-treated THP-1 cells were detected. The high expression level of CD147 was observed in the differentiated process. Zymography, RT-PCR and ELISA results showed that the secretion and activation of MMP-2 and MMP-9 were significantly enhanced in the differentiated THP-1 cells. MMP-9 mRNA expression significantly increased in the differentiated THP-1 cells. Our results agree with the reports that the stimulation of the cells by PMA significantly augmented the release of MMP-9 [19,20].

In view of the fact that CD147 may be required for the expression of gelatinases MMP-2 and MMP-9 and that the gelatinases MMP-2 and MMP-9 are expressed in leukemic cells [19,20,26], our study focused on the potential interaction between CD147 and MMPs, MMP-2 and MMP-9, in the differentiation process of monocytes into macrophages and in the invasion of monocytes/macrophages. We found that the overexpression of CD147 induced elevated levels of proMMP-2, pro-MMP-9 and their activated forms in differentiated THP-1 cells and the elevated levels of MMPs in turn enhanced the invasive ability of THP-1 cells. But the elevated expression and activation of MMPs and the enhanced invasive ability of THP-1 cells were blocked when HAb18G/CD147 antigonistic peptide AP-9 was added. These findings indicate that CD147 is involved in promoting the expression and activation of MMPs, which in turn increases the invasive potential of THP-1 cells.

## Conclusion

The results suggest that the expression of CD147 is upregulated during the differentiation of monocyte THP-1 cells to macrophage cells, and CD147 induces the secretion and the activation of MMP-2 and MMP-9 and which in turn enhance the invasive ability of THP-1 cells. The increased secretion and the activation of MMP-2 and MMP-9 and the enhanced invasive ability of THP-1 cells can be blocked by antagonistic peptide of CD147. The matured monocytes / macrophages, via their high expression of CD147, may play an important role in promoting the tissue repair or tissue damage during their inflammatory response. These findings, together with a better understanding of the possible mechanism and regulation of CD147 on MMPs production, will help the development of innovative therapeutic intervention for inflammatory diseases.

## Methods

### Cell culture

The human monocytic THP-1 cells (American Type Culture Collection, Manassas, Va.) were cultured in RPMI 1640 medium supplemented with 10% fetal bovine serum (Gibco BRL, Gaithersburg, Md.), 1% penicillin/streptomycin and 2% L-glutamin at 37°C in a humidified atmosphere of 5% CO_2_. For the induction of cell differentiation, cells (5 × 10^5 ^to 10^6 ^per ml) were seeded in RPMI 1640 serum medium with 200 nM PMA for 24 h [27]. After incubation, nonattached cells were removed by aspiration, and the adherent cells were washed with RPMI 1640 three times. THP-1 cells in RPMI 1640 without PMA were used as control (undifferentiated) cells.

### Flow cytometry analysis

Expression of CD14 and CD147 on the surface of the differentiated and undifferentiated THP-1 cells was determined by flow cytometry. Cells (5 × 10^5^) were washed 3 times with phosphate-buffered saline (PBS) and then were treated respectively with fluorescein isothiocyanate (FITC)-conjugated anti-CD14 antibody (Becton-Dickinson, USA), and FITC-conjugated anti-CD147 antibody (or FITC-conjugated Mouse IgG1, control, R&D, USA), for 20 minutes in a dark condition. Cells were washed with PBS and then analyzed on a FACS Calibur flow cytometry. Data were processed using the Cell Quest software (Becton-Dickinson, USA).

### Reverse transcription-polymerase chain reaction

Using a Fast Track messenger RNA (mRNA) isolation kit (Invitrogen, San Diego, CA), RNA was extracted from the differentiated and undifferentiated cells using Trizol Reagent. The isolated total RNA (3 ug) was reverse transcribed to complementary DNA (cDNA) with a Ready to Go T-Primed First-Strand kit. The completed first-strand cDNA was amplified by PCR with primers specific for MMP9 (sense and reverse). β-actin mRNA was used as an inner parameter. Amplification was performed by adding 25 ul of RNase Free dH_2_O, 5 ul of dNTP mix, 5 ul 10× PCR buffer, and 1 ul of AmpliTaq DNA polymerase. The reaction tubes were heated to 94°C for 2 minutes and then used 28 cycles of 94°C for 30 seconds, 56°C for 60 seconds and 72°C for 90 seconds. The samples were then incubated at 72°C for 7 minutes. PCR products were electrophoresed on 1% agarose gels and visualized by ethidium bromide staining.

### Gel zymography analysis

Zymography of gelatinases, the method not only reveals the activated form of the enzyme but also the zymogen, giving rise to a closely spaced doublet of digestion. When enzymes are electrophoresed in gels where their substrate is co-polymerized with polyacrylamide, they leave traces of their activity under suitable conditions (Gels were incubated first in a solution containing 2 % Tween 80 and 50 mM Tris, pH 7.5, and then in a solution containing additionally 5 mM CaCl_2 _and 1 μM ZnCl_2 _at 37°C in order to remove SDS from the gels and activate gelatinases). Gelatinase activity was revealed by negative staining with Coomassie Brilliant blue and quantified by densitometry [28]. The differentiated and undifferentiated THP-1 cells in serum-free medium were cultured for 5–20 h in the presence or absence of HAb18G/CD147 antagonist peptide AP-9, which was produced and characterized in our lab based on HAb18G. HAb18G is a new member of CD147 family and is abundantly expressed in human hepatoma tissues and on the cell surface of several hepatoma cell lines with a highly invasive potential [29-32]. Conditioned media were collected and the MMP activity was determined by SDS-polyacrylamide gel zymography. Media samples were centrifuged to remove cellular debris and the supernatant was collected and stored at -20°C. Each sample suspension (30 μl) was mixed with SDS sample buffer without reducing agent and loaded onto a 10% polyacrylamide gel containing 0.1% gelatin. After electrophoresis, gels were washed in 2.5% Triton X-100 and incubated in low salt collagenase buffer containing 50 mmol/l Tris, 0.2 mol/l NaCl, 5 mmol/l anhydrous CaCl_2 _and 0.02% Brijdetergent at 37°C for 30 minutes. The gels were subsequently stained with 0.5% Comassie blue (R-250) and destained with buffer consisting of 20% methanol, 10% acetic acid and 70% distilled water for 30 minutes to visualize the zymogen bands. The zymography gels were scanned and analyzed using US National Institutes of Health Image 1.6 software.

### Invasion assay

The chemotactic cell invasion assay was performed using 24-well transwells units (Costar, Cambridge NY, USA), each with an 8-um pore size polycarbonate filter coated with Matrigel (5 ug/ml in cold medium) to form a continuous thin layer. Prior to the addition of cell suspension of differentiated and undifferentiated THP-1 cells (3 × 10^5^) in serum-free medium in the presence or absence of HAb18G/CD147 antagonist peptide AP-9, Meantime an irrelevant peptide SSP, a synthetic epitope peptide of SARS-S-protein, FFSTFKCYGVSA, produced in our lab was used as control. The dried layer of Matrigel matrix was rehydrated with medium without fetal bovine serum (450 ul). The cells were then cultured for 24 h at 37°C in a CO_2 _incubator. The cells remaining in the upper compartment were completely removed with gently swabbing. The number of cells invaded through the filter into the lower compartment was determined using colorimetric HE assay.

## Authors' contributions

JZ performed the studies and drafted the manuscript. PZ, ZNC, JLJ, conceived of the study, and participated in the design the manuscript. All authors read and approved the final manuscript. QZ participated in the writing assistance. ZBW, XYY, HT, NL, YY provided purely technical help.
